# Effects of the Combining Straw Return with Urease Inhibitor on Ammonia Volatilization, Nitrogen Use Efficiency, and Rice Yield in Purple Soil Areas

**DOI:** 10.3390/plants12112071

**Published:** 2023-05-23

**Authors:** Hong Wang, Kelin Hu, Li Yao, Qi Zhang, Chaowen Lin, Haitao Liu, Fuxiang Luo, Honglin Chen

**Affiliations:** 1 Institute of Agricultural Resources and Environment, Sichuan Academy of Agricultural Sciences, Chengdu 610066, Chinalcw-11@163.com (C.L.);; 2 College of Land Science and Technology, China Agricultural University, Beijing 100193, China

**Keywords:** ammonia volatilization, straw incorporation, urease inhibitor, rice yield, fertilizer N use efficiency

## Abstract

Straw return in rice (*Oryza sativa* L.) paddy has been heavily criticized for its potential to influence ammonia (NH_3_) volatilization loss due to irrational fertilizer N application. Therefore, improving the N fertilization strategies within residue straw systems is necessary to reduce N loss from NH_3_ volatilization. This study investigated how the incorporation of oilseed rape straw and the urease inhibitor affected NH_3_ volatilization, fertilizer N use efficiency (FNUE), and rice yields over two growing seasons (2018–2019) in the purple soil region. This study arranged eight treatments combined straw (2, 5, 8 ton ha^−1^, named 2S, 5S, 8S, respectively), with urea or urease inhibitor (UI, 1% NBPT) with three replicates, which included control (CK), UR (Urea, 150 kg N ha^−1^), UR + 2S, UR + 5S, UR + 8S, UR + 2S + UI, UR + 5S + UI, UR + 8S + UI, based on the randomized complete block method. Our results indicated that incorporating oilseed rape straw increased NH_3_ losses by 3.2–30.4% in 2018 and 4.3–17.6% in 2019 than the UR treatment, attributing to the higher NH_4_^+^-N content and pH value within floodwater. However, the UR + 2S + UI, UR + 5S + UI and UR + 8S + UI treatments reduced NH_3_ losses by 3.8%, 30.3%, and 8.1% in 2018 and 19.9%, 39.5%, and 35.8% in 2019, separately compared to their corresponding UR plus straw treatments. According to the findings, adding 1% NBPT significantly decreased NH_3_ losses while incorporating 5 ton ha^−1^ oilseed rape straw. Furthermore, adding straw, either alone or in conjunction with 1% NBPT, increased rice yield and FNUE by 0.6–18.8% and 0.6–18.8%, respectively. Otherwise, NH_3_ losses scaled by yield in the UR + 5S + UI treatment decreased significantly between all treatments in 2018 and 2019. These results suggest that optimizing the oilseed rape straw rate combined with 1% NBPT applied with urea efficiently increased rice yield and reduced NH_3_ emissions in the purple soil region of Sichuan Province, China.

## 1. Introduction

Nitrogen (N) fertilizer is vital in crop growth and contributes to high crop production. Urea has been frequently applied globally as the N fertilizer due to its high N content (46%), cost-effectiveness per N unit, significant availability, poor corrosion, favorable water solubility, large foliar consumption, and good fertilizer compatibility [1,2]. Rice has been the major cereal crop, feeding more than half of the world’s population, and China ranks first worldwide among rice-producing countries [3]. However, the fertilizer nitrogen use efficiency (FNUE) is very low and only 20–30% in rice fields, attributed to the N loss through nitrate leaching, nitrous oxide emission, and ammonia (NH_3_) volatilization [4]. The volatilization of NH_3_ following urea use has been identified as a main pathway of N loss. Additionally, NH_3_ is known to be the primary atmospheric contaminant, which seriously affects the generation of secondary organic aerosols [5,6]. After emission, NH_3_ is returned to the surface water and lands by deposition, leading to soil acidification, biodiversity loss, and water eutrophication [7,8]. The Sichuan Basin is the most important grain-producing region in Southwestern China. Nitrogen loss from NH_3_ volatilization accounts for 10–43% of the N applied to rice fields in the Sichuan Basin [9]. Purple soil farmland covers 4.06 million ha, accounting for 36.5% of the total farmland in Sichuan Province [10]. Purple soils are unique in China and are used extensively in crop production as the main cropland in the Sichuan Basin. Therefore, optimal management strategies for reducing NH_3_ loss in paddy fields could improve FNUE and crop production, especially in purple soil. 

Currently, different agricultural management strategies help improve rice yield and decrease volatilization of NH_3_, including water management [11,12], cultivation [13], fertilization management [14,15], and the use of exotic substances, such as crop straw or biochar [16,17]. Over the last several decades, straw is usually removed from the field as a construction material or fuel in various countries [18,19]. However, due to excessive fertilizer application, global agricultural lands are increasingly vulnerable to pest attacks and soil agglomerate hardening. Thus, it can promote the return of straw to the field after harvesting since this can enhance soil erosion control, maintain soil moisture, increase soil C stocks, improve the abundance of the bacterial community, and mitigate the volatilization of NH_3_ [20,21,22,23,24]. It is not clear whether straw incorporation can stimulate or suppress NH_3_ volatilization in paddy fields. Some studies found cumulative NH_3_ volatilization fluxes increased with increasing straw incorporation and urea application due to enhanced urea hydrolysis and NH_4_^+^-N concentrations in floodwater [24,25,26], while other studies revealed that tillage management of straw incorporation could reduce NH_3_ volatilization depending on the straw C/N ratio and soil property [27,28]. Tian et al. [29] found that when rice straw was amended at 1500 kg ha^−1^ during three rice seasons, NH_3_ emissions increased by 5.3–22.2% compared to the non-rice straw treatment in the basal fertilization stage. A high volume of straw returned to the soil, one of the effective methods to improve the use of agricultural resources efficiency in the rape-rice rotation system, when considering the effective use of straw resources [17,30]. This highlights the importance of comprehensively investigating the response of NH_3_ volatilization in rice fields due to oilseed rape straw incorporation, particularly in purple soil. 

N-(n-butyl) thiophosphorictriamide (NBPT), a urease inhibitor, helps mitigate NH_3_ volatilization while controlling soil N dynamics. It is the structural analog of urea, rapidly transformed into N-(n-butyl) phosphoric triamide (BNPO), and generates the tridentate ligand with the urease enzyme to compete with the active sites of urease to reduce the hydrolysis of urea [31]. Using urease inhibitors combined with urea can postpone urea hydrolysis while maintaining N in the stable ammonium (NH_4_^+^) form. Therefore, plants can absorb energy-efficient N and reduce N loss through NH_3_ volatilization [32,33]. NBPT has been used to reduce NH_3_ volatilization in winter wheat and rice fields under different soil types [34]. The addition of NBPT improved urea efficiency significantly by increasing plant dry weight, FNUE, and crop production [35]. Furthermore, NBPT significantly inhibits urea hydrolysis, influencing nitrification indirectly [36]. Similarly, urease inhibitors and urea help plants absorb N in the energy-efficient urea or NH_4_^+^ form [37]. The separated use of straw or inhibitors can specifically affect soil NH_3_ volatilization. However, their integrated application can show tradeoffs in NH_3_ volatilization, N transformation, and rice production. Nonetheless, it remains unclear mainly about the comprehensive influences of combined straw incorporation with NBPT in paddy fields on FNUE, NH_3_ volatilization, and grain yield in purple soil. Appropriate measures must be further explored to reduce NH_3_ volatilization and improve FNUE while increasing rice production.

This study used a two-year paddy field experiment to explore how the incorporation of oilseed rape straw and the urease inhibitor affected the volatilization of NH_3_ in the purple soil region of Sichuan Province, China. Therefore, the objectives of this study were to: (1) analyze the effects of oilseed rape straw along with urease inhibitor on NH_3_ volatilization fluxes; (2) quantify the relationship between the variation of NH_4_^+^, NO_3_^−^, pH in floodwater with NH_3_ volatilization; (3) determine the optimal usage of oilseed rape straw associations with a fixed ratio of urease inhibitor.

## 2. Results

### 2.1. NH_3_ Volatilization

**Figure 1:**
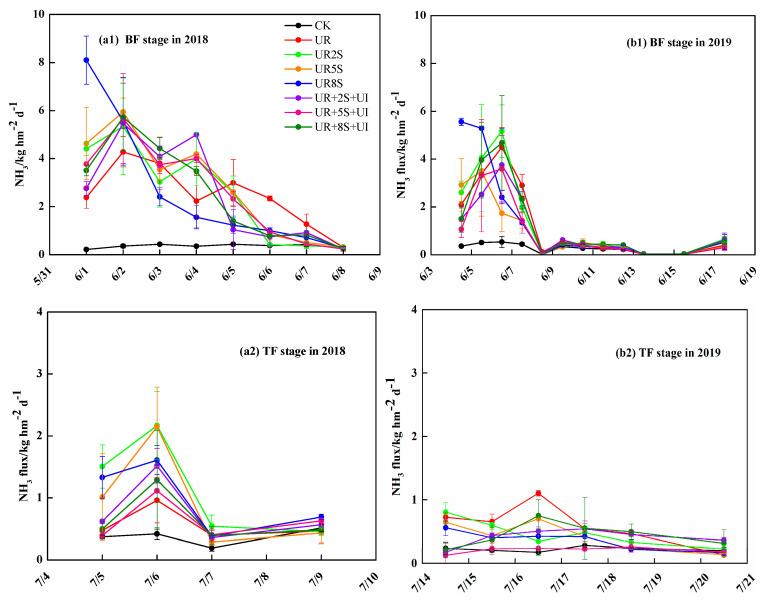

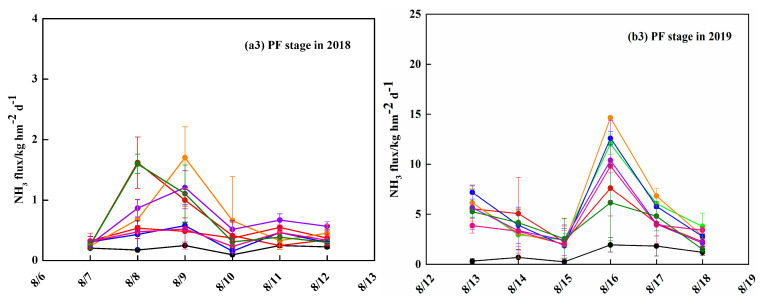
Dynamics of NH_3_ fluxes in the different treatments at the stages of basal (1), tillering (2), and panicle (3) after fertilization in 2018 (**a1**–**a3**) and 2019 (**b1**–**b3**) in rice season ((**a1**,**b1**), BF; (**a2**,**b2**), TF; (**a3**,**b3**), PF).

The temporal variations of NH_3_ volatilization flux based on different treatments after applying basal fertilizer (BF), tillering fertilizer (TF), and panicle fertilizer (PF) in the 2018–2019 rice-growing seasons can be seen in Figure 1. During both the years of the experiment, NH_3_ fluxes elevated after applying urea N fertilizer relative to control. Compared to urea N fertilizer alone, the treatments combined oilseed rape straw with urea significantly increased NH_3_ fluxes after the application of BF, TF, and PF. However, the fluxes of NH_3_ were decreased while increasing the oilseed rape straw from 5 ton ha^−1^ to 8 ton ha^−1^ after TF and PF application in 2018 and TF application in 2019. This can be attributed to the high adsorption capacity of oilseed rape straw of NH_4_^+^ and the gross nitrification enhanced with the increase in oilseed rape straw amounts. Moreover, the flux of NH_3_ drastically increased after applying PF application in 2019 relative to BF, TF, and PF application in 2018 and BF and TF application in 2019. Our result concerns the increase in the flux of NH_3_ to an increase in air temperature.

The volatilization flux of NH_3_ after applying BF, TF, and PF was influenced by oilseed rape straw combined with 1% NBPT. The daily NH_3_ fluxes were reduced significantly in the UR + 5S + UI treatment after fertilizer application relative to the UR + 5S treatment (*p* < 0.05). Those highest peaks occurred two or three days after application and decreased rapidly 4–5 days later. The oilseed rape straw treatments produced the highest peaks for one or two days, but the addition of 1%NBPT treatments delayed the peaks by several days.

The N loss by NH_3_ volatilization under different treatments in the 2018–2019 rice-growing season is shown in Table 1. The overall NH_3_ losses under UR treatment in 2018 and 2019 were 28.7 and 47.7 kg N ha^−1^, respectively. Compared to UR treatment, the addition of oilseed rape straw (UR + 2S, UR + 5S, and UR + 8S) increased NH_3_ losses by 3.2% to 30.4% in 2018 and 4.3% to 17.6% in 2019, respectively. The total NH_3_ losses under the UR + 2S + UI, UR + 5S + UI, and UR + 8S + UI treatments were 29.1, 26.1, and 27.2 kg N ha^−1^ in 2018, decreased by 3.8%, 30.3%, and 8.1% compared with those from the UR + S (UR + 2S, UR + 5S, and UR + 8S) treatments, respectively. While they are 39.9, 32.8, and 47.2 kg N ha^−1^ in 2019, decreased by 19.9%, 39.5%, and 16.0% than their corresponding UR + S (UR + 2S, UR + 5S, and UR + 8S) treatments, respectively, which are much higher than their decrease ranges in 2018. The NH_3_ losses mainly occurred following BF and PF application which occupied 72–97% of the NH_3_ losses over the two years due to significant N inputs, high temperature, and poor N absorption through rice seedlings. Meanwhile, cumulative NH_3_ emissions increased after PF application compared to BF for all treatments in 2019. The addition of oilseed rape straw promoted the NH_3_ emission factor during the two growing seasons. In comparison, the UR + 5S + UI treatment exhibited a minor NH_3_ emission factor, significantly decreasing from 19.3% to 13.5% in 2018 and from 29.6 to 15.3% in 2019 compared to the UR + 5S treatment.

### 2.2. Dynamic of Inorganic N Concentration and pH in Floodwater

**Figure 2:**
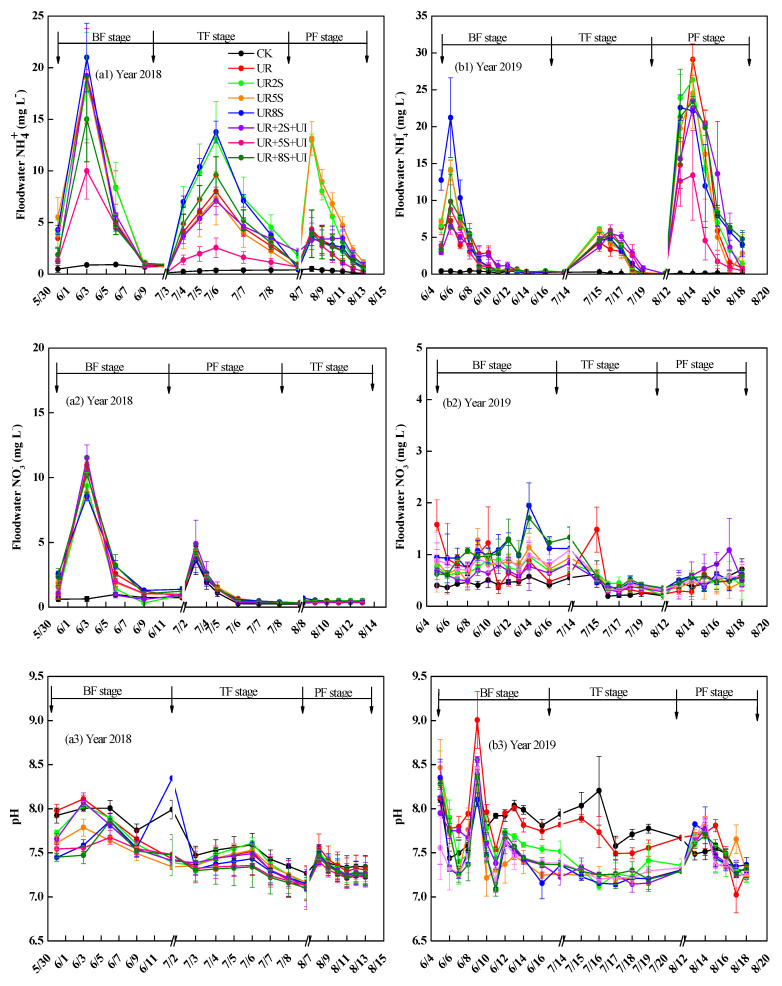
Dynamics of floodwater NH_4_^+^−N and NO_3_^−^-N concentrations and pH in the different treatments ((**a1**,**b1**), floodwater NH_4_^+^ concentration; (**a2**,**b2**) floodwater NO_3_^−^ concentration; (**a3**,**b3**), pH).

Temporal changes in pH, NH_4_^+^-N, and NO_3_^−^-N concentrations in floodwater under various treatments during the 2018–2019 rice seasons are shown in Figure 2. The NO_3_^−^-N and NH_4_^+^-N contents within floodwater showed an increasing trend after UR treatment relative to control and exhibited a decreasing tendency to the range close to control on days 9–15. The NH_4_^+^-N contents within UR floodwater were 3.21 and 6.06 mg N L^−1^ on average in 2018 and 2019, respectively. Compared to the UR treatment, the UR + 2S treatment increased the contents of NH_4_^+^ in floodwater after BF and PF applications in 2018 (*p* < 0.05), while they remained unchanged after fertilizer application in 2019. Meanwhile, the NH_4_^+^-N contents in the floodwater under UR + 8S treatment were 4.14 and 6.3 mg N L^−1^ on average in 2018 and 2019, which was reduced by 70.8% and 4.1% relative to that under UR + 5S treatment. The NH_4_^+^-N contents in the floodwater under UR + 2S + UI, UR + 5S + UI, and UR + 8S + UI treatments were 5.4, 3.7, and 3.4 mg N L^−1^ in 2018, whereas 3.9, 3.4, and 5.0 mg N L^−1^ in 2019, decreased by 18.7%, 47.2% (*p* < 0.05), and 17.3% in 2018, 30%, 48.3%, and 20.4% in 2019 (*p* < 0.05) than those from the UR + S (UR + 2S, UR + 5S, and UR + 8S) treatments, respectively. Overall, NO_3_^−^-N contents in floodwater did not change significantly after applying fertilizers under all treatments. However, UR + 8S treatment increased (*p* < 0.05) floodwater NO_3_^−^-N content after basal application compared with the UR treatment.

Floodwater pH ranged between 7.3–7.9 in 2018 and 7.2–7.9 in 2019. The addition of oilseed rape straw alone resulted in an additional reduction of floodwater pH, and a large amount of oilseed rape straw resulted in lower pH values. Floodwater pH was reduced in the UR + 5S + UI and UR + 8S + UI treatments and was lower than those in the UR, UR + 5S, and UR + 8S treatments (*p* < 0.05). Compared to UR treatment, the floodwater pH of UR + 5S + UI and UR + 8S + UI treatments was lower by 0.13 in 2018 and 0.29 in 2019, respectively.

### 2.3. Rice Yields, FNUE, and Yield-Scaled NH_3_ Volatilization

The rice yields under UR treatment were 5.24 ton ha^−1^ in 2018 and 6.77 ton ha^−1^ and 2019, separately (Table 2). Compared to the control, the UR treatment increased rice yield by 32.0% in 2018 and 34.6% in 2019. Moreover, compared with UR, the rice yield increased by 5.9%, 12.8%, 0.8%, 7.8%, 19.7%, 15.8% in the UR + 2S, UR + 5S, UR + 8S, UR + 2S + UI, UR + 5S + UI, and UR + 8S + UI treatments in 2018, while they increased by 3.6%, 1.3%, 10.0%, 0.8%, 9.4%, and 5.8% in 2019, which are much lower than their increase ranges in 2018. Application of oilseed rape straw alone or in combination with 1% NBPT elevated N absorption through the rice plants in 2018, and more oilseed rape straw led to higher N uptake. During the UR treatment, the FNUE was 34.9 kg kg^−1^ and 41.3 kg kg^−1^ in 2018 and 2019, respectively, 0.6–18.8% lower than those in the UR + 2S, UR + 5S, UR + 8S, UR + 2S + UI, UR + 5S + UI, and UR + 8S + UI treatments. However, using 1% NBPT with urea and more oilseed rape straw increased FNUE and rice yield slightly but not significantly. Rice yield was significantly associated with year, UR, UR + 5S, UR + 8S, UR + 5S + UI, and UR + 8S + UI (*p* < 0.01, Table 3).

**Figure 3:**
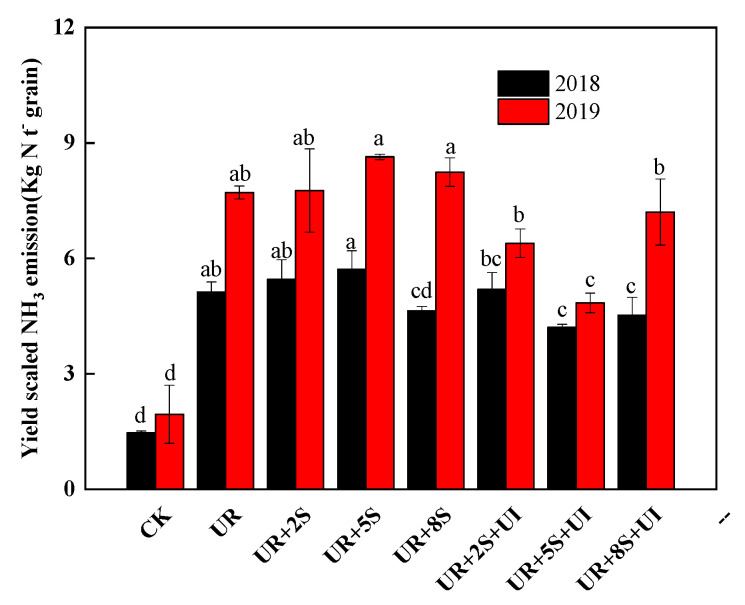
Effects of oilseed rape straw and 1% NBPT on yield-scaled NH_3_ volatilization from the treatments in 2018 and 2019. Bars are standard errors (*n* = 3). Different letters indicate significant differences between treatments within the same year (*p* < 0.05).

The yield-scaled NH_3_ emissions under UR + 2S and UR + 8S treatments decreased with increasing rape straw amount in 2018, while all the UR + S treatments increased the yield-scaled NH_3_ emissions by 0.7–12.0% in 2019 compared to the UR treatment (Figure 3). The yield-scaled NH_3_ emissions under the UR treatment were 5.12 and 7.71, which was lower by −1.4%, 17.7%, and 11.7%, in 2018, and 17.1%, 37.2%, and 6.6% in 2019, separately, compared to the UR + S treatment. The reduction range in 2019 was greater than that in 2018. Compared to the UR + S treatments, the yield-scaled NH_3_ emissions under UR + 2S + UI, UR + 5S + UI, and UR + 8S + UI treatments decreased by 4.8% and 17.7%, 26.3% in 2018 and 44.0%, and 2.5% and 12.6% in 2019, respectively. Furthermore, the UR + 5S + UI treatment had the greatest reduction (*p* < 0.05).

## 3. Discussion

### 3.1. The Effect of Straw Incorporation on NH_3_ Volatilization

It remains unclear mainly how straw affects NH_3_ volatilization, inhibition [29,38,39], and stimulation [24,26] have been variously reported. Wang et al. [24] found that after the incorporation of wheat straw at 6.5 ton ha^−1^, NH_3_ volatilization showed an increase of 28.5% relative to non-straw incorporation. Sun et al. [26] reported that 8 ton ha^−1^ rice straw application significantly expedited the 8.1% of NH_3_ volatilization compared to the control. According to the results of over a two-year average in our study, the cumulative volatilization of NH_3_ increased with increasing the amount of oilseed rape straw during the rice growing season. Our results agreed with the findings reported by Wang et al. [24] and Sun et al. [26]. There are two main reasons for that. Firstly, it increased soil microbe activity and improved urea hydrolysis while increasing NH_4_^+^-N content within floodwater [40]. Secondly, an increase in NH_3_ volatilization is associated with urease within straw and higher floodwater pH [24,26]. Sun et al. [26] also showed that straw decomposition increased energy release, urease activity, and soil temperature. However, our investigation showed that NH_3_ volatilization was reduced with oilseed rape straw incorporated at 8 ton ha^−1^ compared to 5 ton ha^−1^ after BF and TF in 2018 and TF and PF in 2019. This is because there was poor immobilization and decomposition of N released after incorporating C-abundant materials [41]. According to Tian et al. [29], urea combined with rice straw reduced NH_3_ volatilization in wheat fields during the stem elongation period. Liu et al. [39] found the chemical fertilizer with straw return treatment inhibited the loss of NH_3_ by 22.7–24.1% compared to the chemical fertilizer treatment. Philippe et al. [38] also observed straw mulching in no-till fields reduced NH_3_ volatilization by 82.7% N loss to till soils, due to changes in the soil micro-environment caused by straw mulching. Our results disagreed with the findings because their results were obtained from dryland.

### 3.2. The Effect of Urease Inhibitor on NH_3_ Volatilization

Cumulative volatilization decreased under 1% NBPT amended treatments compared to the oilseed rape straw and UR treatments. The NH_3_ cumulative emissions were reduced by 13.8%, 35.8%, and 13.3% for the UR + 2S + UI, UR + 5S + UI, and UR + 8S + UI treatments relative to UR + S treatments, respectively, whereas they decreased by 9.7%, 23.0%, and 2.7% compared to UR treatment, respectively, over a two-year average. Results showed that UR + 5S + UI treatment was more effective in reducing NH_3_ volatilization compared with UR + 2S + UI and UR + 8S + UI treatments. Our findings are consistent with previous reports, which observed that adding NBPT to urea significantly suppressed soil activity and postponed the hydrolysis of urea within the soil [33,42,43,44,45,46,47]. For instance, Regina et al. [46] revealed that NH_3_ losses were reduced by 70% after applying urea plus NBPT. Our results highlight that these NH_3_ losses were reduced due to high NO_3_^−^-N concentrations in flood water caused by the addition of oilseed rape straw and 1% NBPT after fertilizer application.

Rice yields were increased by adding oilseed rape straw and urea N fertilizer compared to UR and control treatments. Zeng et al. [48] indicated that straw positively affected the increased productivity of agriculture by 1.7–145.8% due to the improved soil properties. Su et al. [49] observed increased yields of oilseed rape because of the elevated soil N and water availability and reduced soil temperature fluctuations. However, applying oilseed rape straw with and without 1% NBPT slightly increased FNUE and rice production but was insignificant. Similar results have been described by Cantarella et al. [2], who state that reduced NH_3_ volatilization, had a significantly limited impact on rice yield. According to Liu et al. [50], using UI to reduce NH_3_ loss for N conservation had no significant effect on grain yield.

The combination of 2 ton ha^−1^ and 8 ton ha^−1^ oilseed rape straw and 1% NBPT slightly reduced yield-scaled NH_3_ emissions, while the yield-scaled NH_3_ emissions for UR + 5S + UI treatment were reduced more significantly than other treatments. The addition of oilseed rape straw increased rice yield compared to UR treatment with 1% NBPT, while the efficiency of NBPT was low in UR + 8S + UI treatment due to the application of the largest oilseed rape straw amount [51]. Our results demonstrated that 1% NBPT combined with urea at 5 ton ha^−1^ or the total amount of rape straw in the field effectively increased FNUE and rice yield. Furthermore, it also reduced NH_3_ emission at the yield scale during rice growing seasons in purple soil areas of Southwestern China.

### 3.3. The Effect of Other Factors on NH_3_ Volatilization

Climate conditions, such as air temperature and wind speed, affect NH_3_ fluxes [38,40]. Furthermore, volatilization of NH_3_ could migrate from the soil in rain or lower temperature conditions [48,52]. Thus, floodwater and air temperatures in the PF stage reduced in 2018 than in 2019. In addition, more frequent rain was observed in the TF and PF stages in 2018 than in 2019. The persistent rain resulted in a decrease in the NH_4_^+^-N content in the floodwater and heavy rainfall on day two following PF, thus, causing the floodwater to overflow from the field of the experiment in 2018. Consequently, 2018 had significantly reduced fluxes of NH_3_ volatilization compared to 2019.

Based on the two-year field trial, NH_4_^+^-N contents in floodwater increased in straw-incorporated plots compared to the non-straw incorporation. Therefore, the NH_3_ fluxes depicted a relation with the NH_4_^+^-N content within floodwater, where the NH_4_^+^-N content in floodwater increased after the oilseed rape straw treatment compared to the treatment with UR after fertilizer application. Daniel et al. [16] found that adding 10 ton ha^−1^ rice straw to paddy soil enhanced fertilizer N immobilization in paddy soils. Increased NO_3_^–^-N contents were detected in the UR + 8S treatment in 2019 compared to the UR treatment via oilseed rape straw-enhanced soil nitrification. Ren et al. [53] proposed that crops compete for N with soil microorganisms, which explains the higher N gross immobilization rate. According to Liu et al. [54], the higher ammonium level caused by amended straw decomposition provided more nitrification substrate via ammonia oxidizers than non-straw treatments. In this study, NH_3_ flux was associated with floodwater pH, correlating with prior reports [55,56]. This study showed that applying oilseed rape straw reduced floodwater pH after BF in 2018 and TF in 2019. Our findings were supported by a previous pH reduction of 7.1 ± 0.03–5.4 ± 0.09 of NPK fertilizers relative to non-fertilization treatment, regardless of low or high wheat straw content [26]. This is possible due to the generation of certain acidic substances during the degradation of cellulose [57,58,59]. Furthermore, Soares et al. [43] pointed out that reduced floodwater pH due to nitrification caused by the high NO_3_^−^-N content within soil and floodwater after UR and UR + NBPT treatments after fertilizer application.

Based on our results, NH_3_ was volatilized within paddy soil during the 1-week fertilization in the flux patterns, regardless of the straw amendment. The N loss due to NH_3_ volatilization is quantifiable. On the contrary, Huang et al. [9] found that the average cumulative NH_3_ emissions in paddy fields in the same area of China were lower than 67.5 kg N ha^−1^. It is primarily associated with the urea-N used in the surface broadcast of basal, tillering, and panicle fertilizers rather than the one-time rice fertilizer. According to Liu et al. [54], farmers typically used one-time rice BF for convenience, which increased the NH_4_^+^-N level in floodwater and the surficial soil layer and could increase the risk of N losses. Another reason is that rice roots are small, and plants have low N demand, which increases the risk of N loss during basal fertilization [12]. Furthermore, the rice jointing-booting stage had the maximal N absorption rate, while sufficient N supply in this period improved rice production [60]. In the present study, the emission factor for N application treatments was 19.0% over a two-year average, which was higher than 15.6% reported by He et al. [56]. Therefore, fertilizer N application in purple soil for rice production has a greater potential for NH_3_ loss reduction.

## 4. Materials and Methods

### 4.1. Experimental Site

**Figure 4:**
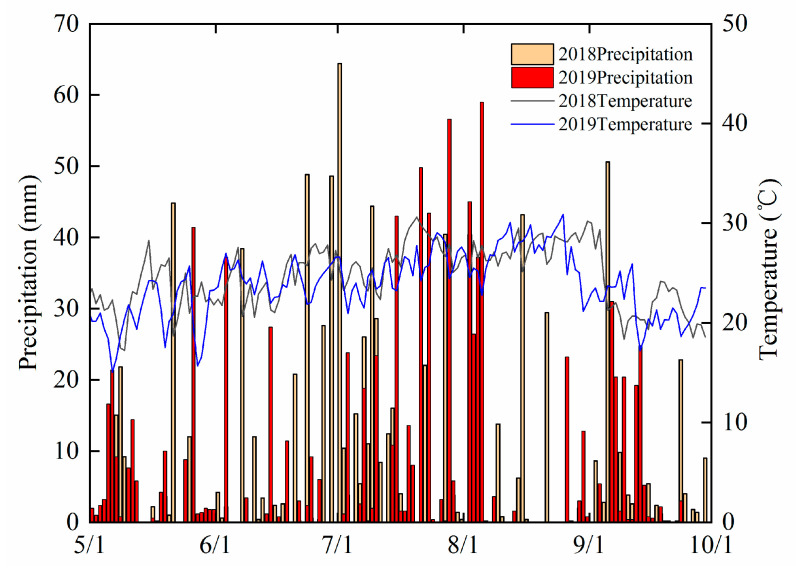
Variations of air temperature and precipitation during rice growth.

The field experiment was conducted at the Ziyang Experimental Station of Sichuan Academy of Agricultural Sciences located in the upper reaches of Tuojiang River, a major branch of the Yangtze River (104°32′12″–104°35′19″ E; 30°05′12″–30°06′44″ N; elevation, 395 m). This site has a subtropical monsoon climate, with the average annual temperature being 16.8 °C. January and July are the coldest and warmest, and the average minimal and maximum temperatures are 7.4° C and 27.4 °C, respectively. Furthermore, the average annual precipitation is 966 mm, with 70% of the falls received during June–September. According to US Taxonomy, Purple soil is the most common soil type categorized into Entisol. It is typically found at a depth of 50–80 cm, with low soil productivity and a light texture. The average precipitation and daily air temperature for the two rice-growing seasons are shown in Figure 4.

### 4.2. Treatments and Experiments

The field experiment was initiated in May 2018. The experiment consisted of two factors, one factor being the amounts of oilseed rape straw (0, 2, 5, 8 tons of dry matter (DM) ha^−1^) and the other being the fixed amount of urease inhibitor, at a supply rate of 1%. The arrangement resulted in eight different treatments with three duplicates as shown in Table 4. Each plot is 5 m × 6 m and the randomized block design was adopted. The NBPT (Agrotain Ultra, Koch Fertilizer, LLC, Wichita, KS, USA) was applied by hand to urea at a rate of 10 g NBPT kg^−1^ urea.

The tested rice (*Oryza sativa* L.) variety was Yixiang 2115 in both years. The rice was transplanted on 30 May 2018, and on 2 June 2019, at 20 cm × 24 cm spacing; meanwhile, the BF was applied, including 60% of total N as urea (i.e., 60 kg N ha^–1^), 105 kg K_2_O ha^–1^ and 75 kg P_2_O_5_ ha^–1^. This experiment also used TF (30 kg N ha^–1^) and PF (60 kg N ha^–1^) on 4 July, 7 August in 2018, and 14 July, and 12 August in 2019, separately. It produced a total N input of 150 kg N ha^–1^ during the rice growing season, where each column was within an equivalent irrigation level (about 5 cm flood water level). The rice was harvested on 16 September 2018 and on 20 September 2019. At the harvesting stage, straw and grain separately collected from experimental plots in each replication were used to measure the yields. Plants from 10 rice plants from each subplot were randomly sampled at the harvesting stage to study the moisture content and N uptake differences between the fertilization patterns. Rice grain moisture content reported herein is 16.4–19.6%, and rice straw moisture content is 65.1–69.9%. Urea was broadcast to the field. The straw utilized in this work was derived from the harvested oilseed rape crop. One week before rice transplantation, the oilseed rape straw was air-dried, cut into 5 cm pieces, and mixed into topsoil (0–20 cm). The total N, organic C, total P, and total K contents of oilseed rape straw were 0.42%, 40.6%, 0.071%, and 1.85%, respectively.

### 4.3. Determination of NH_3_ Volatilization

The continuous air flow enclosure approach using the dynamic plexiglass cylindrical chamber (height, 18 cm; inner diameter, 25 cm) could measure NH_3_ volatilization loss [61]. The acrylic chamber was also placed in the soil at 12 cm to prevent air flux on the soil surface. Daily fluxes in NH_3_ volatilization were determined daily between 8:00–10:00 am and 15:00–17:00 pm [12]. After every fertilizer application, daily NH_3_ fluxes were measured between 31 May–9 June, 5–9 July, 7–12 August 2018, 5–18 June, 15–21 July, and 13–18 August 2019.

Volatilized NH_3_ was trapped by a gas washing bottle containing 60 mL of 20% (*w*/*w*) boric acid. After every measurement, this work eliminated the chamber from the soil to minimize the heterogeneities of climate conditions in and out of the chamber. On each sampling date, the sorbers were removed, then the NH_3_ concentration was determined using 0.01 mol L^−1^ HCl, with the bromocresol green–methyl red mixture within ethanol being the indicator. The NH_3_ flux was calculated by using Equation (1):

(1) $$F_{av}=\frac{M}{A\cdot D}\times 10^{-2}$$

where *F_av_* is the NH_3_ flux rate (kg N ha^−1^ d^−1^), *M* is the collected amount of NH_3_ by the cylindrical chamber (mg), *A* is the cross-sectional area of the cylindrical chamber (m^2^), *D* is the interval of collection (days).

In addition, the NH_3_ emission factor, the yield-scaled NH_3_ volatilization, and the fertilizer nitrogen use efficiency (FNUE) were calculated by using Equation (2), Equation (3), and Equation (4), respectively.

(2) $${NH}_{3}\;\mathrm{emission}\;\mathrm{factor}=\frac{E_{f}-E_{c}}{N_{f}}$$

(3) $$\mathrm{Yield}-\mathrm{scaled}\;{NH}_{3}\;\mathrm{emission}=\frac{E_{f}}{Y}$$

(4) $$\mathrm{FNUE}=\frac{Y}{N_{f}}$$

where *E*_f_ and *E*_c_ are the total cumulative NH_3_ volatiliztion from the N fertilizer and control treatments (kg N ha^−1^), respectively. *N*_f_ is the fertilizer N rate (kg N ha^−1^). Y denotes the grain yield (kg ha^−1^) under each treatment.

### 4.4. Floodwater Sample Collection and Analyses

During the period of NH_3_ volatilization measurement, the pH and temperature of the floodwater were recorded. Floodwater was collected randomly from five locations and thoroughly mixed into the combined sample. When determining NH_3_ volatilization, floodwater samples were taken at one-day intervals, brought to the laboratory, and analyzed within 2 h using the Skalar segmented flow analyzer (Skalar, Breda, The Netherlands). Meteorological data were collected from a meteorological station near the study site from 2018 to 2019.

### 4.5. Statistics Analysis

The current work used the least significant difference (LSD), and standard analysis of variance (ANOVA) tests at a significance level of 5% to identify differences between the incorporation of oilseed rape straw and the incorporation of 1% NBPT in volatilization of NH_3_, FNUE, rice production, and emission factor. The covariance analysis (ANCOVA) examined the possible influences of urea fertilizer, year, straw, urease inhibitor, and the relationships between rice production and NH_3_ volatilization. IBM SPSS Statistics 26 (SPSS, IBM, Armonk, NY, USA) and R version 3.0.2 were used for statistical analysis.

## 5. Conclusions

The field study assessed the integrated role of oilseed rape straw and 1% NBPT in NH_3_ volatilization, FNUE, and rice yield in the two seasons. An amount of 2 ton ha^−1^ oilseed rape straw stimulated NH_3_ emissions in the two years, and NH_3_ fluxes increased with increasing oilseed rape straw. The combined use of oilseed rape straw in the 2–5 ton ha^−1^ range with 1% NBPT could substantially reduce NH_3_ losses. In contrast, 8 ton ha^−1^ straw with 1% NBPT increased NH_3_ losses. Surprisingly, the co-application of 1% NBPT and urea to 5 ton ha^−1^ oilseed rape increased FNUE and rice yield while decreasing yield-scaled NH_3_ emissions. As a result of the integrated influence on agricultural production in the current paddy field study, straw incorporation and UI effectively optimize production while decreasing NH_3_ emissions. Combining approximately 5 ton ha^−1^ oilseed rape straw or the total amount of oilseed rape straw in the field with 1% NBPT effectively promoted the economic and environmental benefits of reduced NH_3_ emission and improved rice production in the purple soil area of Southwestern China.

## Figures and Tables

**Table 1:** Mean ammonia emissions and ammonia emission factors following N fertilizer application and across the two years of the study in the rice season.

Year	Treatment	NH_3_ Emission (kg N ha^−1^)				NH_3_ Emission Factor (%)			
		BF	TF	PF	Total	BF	TF	PF	Total
2018	CK	3.0 c	1.6 d	1.3 d	5.8 c				
	UR	23.0 b	2.9 c	2.8 bc	28.7 b	33.3 ab	4.3 c	2.6 bc	15.2 ab
	UR + 2S	21.2 b	5.9 a	3.2 bc	30.3 ab	30.3 ab	14.3 a	2.4 bc	16.0 ab
	UR + 5S	27.2 a	5.0 b	5.2 a	37.4 a	40.3 a	11.2 b	2.4 c	19.3 a
	UR + 8S	21.1 b	4.3 b	4.2 ab	29.6 ab	30.2 ab	8.9 b	5.0 a	15.8 ab
	UR + 2S + UI	22.6 ab	2.6 c	3.9 ab	29.1 ab	32.7 ab	2.9 c	4.4 ab	15.4 ab
	UR + 5S + UI	21.1 b	2.6 c	2.3 cd	26.1 b	30.3 ab	3.4 c	1.7 c	13.5 b
	UR + 8S + UI	20.7 b	2.7 c	3.8 abc	27.2 b	29.4 b	3.8 c	5.7 a	14.8 b
2019	CK	3.6 c	1.5 c	4.7 b	9.8 c				
	UR	12.0 ab	4.1 a	31.7 a	47.7 a	14.0 ab	8.5 a	45.0 bc	25.3 ab
	UR + 2S	16.2 ab	3.2 ab	33.4 a	49.8 a	16.0 ab	5.7 ab	47.8 abc	26.7 ab
	UR + 5S	11.5 ab	2.2 bc	40.5 a	54.2 a	13.2 ab	2.4 c	59.6 a	29.6 a
	UR + 8S	19.9 a	1.9 c	38.5 a	56.1 a	20.2 a	1.4 c	56.3 ab	30.9 a
	UR + 2S + UI	9.1 b	2.7 bc	28.1 ab	39.9 a	9.2 b	4.0 bc	39.0 cd	20.1 bc
	UR + 5S + UI	8.7 b	1.9 c	22.2 ab	32.8 b	8.5 b	1.3 c	29.2 d	15.3 d
	UR + 8S + UI	11.3 ab	1.7 c	34.1 a	47.2 a	12.9 ab	0.8 c	38.9 cd	24.9 c
Two-year average	CK	3.3 b	1.6 d	3.0 b	7.8 c				
	UR	17.5 a	3.5 ab	17.3 a	38.2 ab	23.6 a	6.4 ab	23.8 bc	20.3 bc
	UR + 2S	17.2 a	4.5 a	18.3 a	40.0 ab	23.1 a	10.0 a	25.1 bc	21.3 ab
	UR + 5S	19.3 a	3.6 ab	22.9 a	45.8 a	26.7 a	6.8 ab	31.0 a	24.5 a
	UR + 8S	18.4 a	3.1 abc	21.4 a	42.9 a	25.2 a	5.2 abc	30.6 ab	23.4 ab
	UR + 2S + UI	15.9 a	2.7 bcd	16.0 a	34.5 ab	20.93 a	3.4 bc	21.7 c	17.7 bc
	UR + 5S + UI	14.9 a	2.3 cd	12.3 ab	29.4 b	19.4 a	2.4 c	15.5 d	14.4 c
	UR + 8S + UI	16.0 a	2.2 cd	19.0 a	37.2 ab	21.2 a	2.3 c	22.3 c	19.9 bc

BF, basal fertilizer; TF, tillering fertilizer; PF, panicle fertilizer. Different letters in each group indicate significant differences between different treatments (*p* < 0.05).

**Table 2:** Effect of oilseed rape straw and urease inhibitors or rice yield, crop N uptake, and FNUE.

Year	Treatment	Yield (t ha^−1^)	Crop N Uptake (kg N ha^−1^)	FNUE (kg kg^−1^)
2018	CK	3.97 ± 0.01 b	67.3 ± 0.1 b	26.5 ± 0.0 b
	UR	5.24 ± 0.38 a	99.1 ± 0.5 a	34.9 ± 2.6 a
	UR + 2S	5.55 ± 0.49 a	105.4 ± 5.8 a	37.0 ± 3.3 a
	UR + 5S	5.91±0.77 a	107.1 ± 11.7 a	39.4 ± 5.1 a
	UR + 8S	5.28 ± 1.53 a	100.6 ± 29.6 a	35.2 ± 10.2 a
	UR + 2S + UI	5.65 ± 0.41 a	104.5 ± 22.4 a	37.7 ± 2.7 a
	UR + 5S + UI	6.22 ± 0.34 a	116.0 ± 3.0 a	41.5 ± 2.2 a
	UR + 8S + UI	6.07 ± 1.00 a	112.2 ± 11.5 a	40.5 ± 6.7 a
2019	CK	5.03 ± 0.34 b	70.7 ± 4.2 d	33.5 ± 2.3 b
	UR	6.19 ± 0.08 a	106.3 ± 9.7 a	41.3 ± 0.5 a
	UR + 2S	6.41 ± 0.44 a	108.4 ± 9.6 bc	42.7 ± 2.9 a
	UR + 5S	6.27 ± 0.20 a	108.9 ± 3.1 bc	41.8 ± 1.4 a
	UR + 8S	6.81 ± 0.37 a	119.5 ± 7.2 a	45.4 ± 2.5 a
	UR + 2S + UI	6.24 ± 0.33 a	114.6 ± 1.1 abc	41.6 ± 2.2 a
	UR + 5S + UI	6.77 ± 0.32 a	111.1 ± 2.0 abc	45.1 ± 2.1 a
	UR + 8S + UI	6.55 ± 0.64 a	118.1 ± 10.0 ab	43.7 ± 4.3 a

FNUE, fertilizer N use efficiency. Different letters in each group indicate significant differences between different treatments (*p* < 0.05).

**Table 3:** Effect of year, oilseed rape straw (S), urease inhibitor (UI), and their interactions on NH_3_ volatilization and rice yield.

Factors	DF	NH_3_ Emissions (kg N ha^−1^)			Yield (t ha^−1^)		
		**SS**	**F**	P	SS	F	P
Y	1	2489.17	63.20	<0.001	3.37	26.95	<0.001
UR	1	1200.84	30.49	<0.001	2.42	19.32	<0.001
UR + 2S	1	30.06	0.76	0.389	0.02	0.17	0.688
UR + 5S	1	265.74	6.75	0.015	0.69	5.48	0.026
UR + 8S	1	268.65	6.82	0.014	2.02	16.13	<0.001
UR + 2S + UI	1	5.52	0.14	0.711	0.17	1.35	0.255
UR + 5S + UI	1	19.66	0.50	0.486	1.66	13.27	0.001
UR + 8S + UI	1	333.61	8.47	0.007	1.40	11.16	0.002
Error	29	39.38	-	-	0.13	-	-

**Table 4:** Field management for different treatments from 2018 to 2019.

Treatment	CK	UR	UR + 2S	UR + 5S	UR + 8S	UR + 2S + UI	UR + 5S + UI	UR + 8S + UI
Fertilizer (kg N ha^−1^)	0	150	150	150	150	150	150	150
Straw(ton ha^−1^)	0	0	2	5	8	2	5	8
NBPT	0	0	0	0	0	1%	1%	1%

CK, control; UR, 150 kg N ha^−1^ urea; UR + 2S, UR + 5S and UR + 8S, 150 kg N ha^−1^ urea combined with 2, 5, and 8 ton ha^−1^ oilseed rape straw, respectively; NBPT, N-(n-butyl) thiophosphorictriamide; UI, urease inhibitor (1% NBPT).

## Data Availability

Data are contained within the article.
